# Advances in the treatment of invasive fungal disease

**DOI:** 10.1371/journal.ppat.1011322

**Published:** 2023-05-04

**Authors:** Zhuan Zhang, Gerald F. Bills, Zhiqiang An

**Affiliations:** Texas Therapeutics Institute, The Brown Foundation Institute of Molecular Medicine, University of Texas Health Science Center at Houston, Houston, Texas, United States of America; Vallabhbhai Patel Chest Institute, INDIA

## Abstract

With over 300 million severe cases and 1.5 million deaths annually, invasive fungal diseases (IFDs) are a major medical burden and source of global morbidity and mortality. The World Health Organization (WHO) recently released the first-ever fungal priority pathogens list including 19 fungal pathogens, considering the perceived public health importance. Most of the pathogenic fungi are opportunistic and cause diseases in patients under immunocompromised conditions such as HIV infection, cancer, chemotherapy, transplantation, and immune suppressive drug therapy. Worryingly, the morbidity and mortality caused by IFDs are continuously on the rise due to the limited available antifungal therapies, the emergence of drug resistance, and the increase of population that is vulnerable to IFDs. Moreover, the COVID-19 pandemic worsened IFDs as a globe health threat as it predisposes the patients to secondary life-threatening fungi. In this mini-review, we provide a perspective on the advances and strategies for combating IFDs with antifungal therapies.

## Conventional antifungals targets

**Fig 1:**
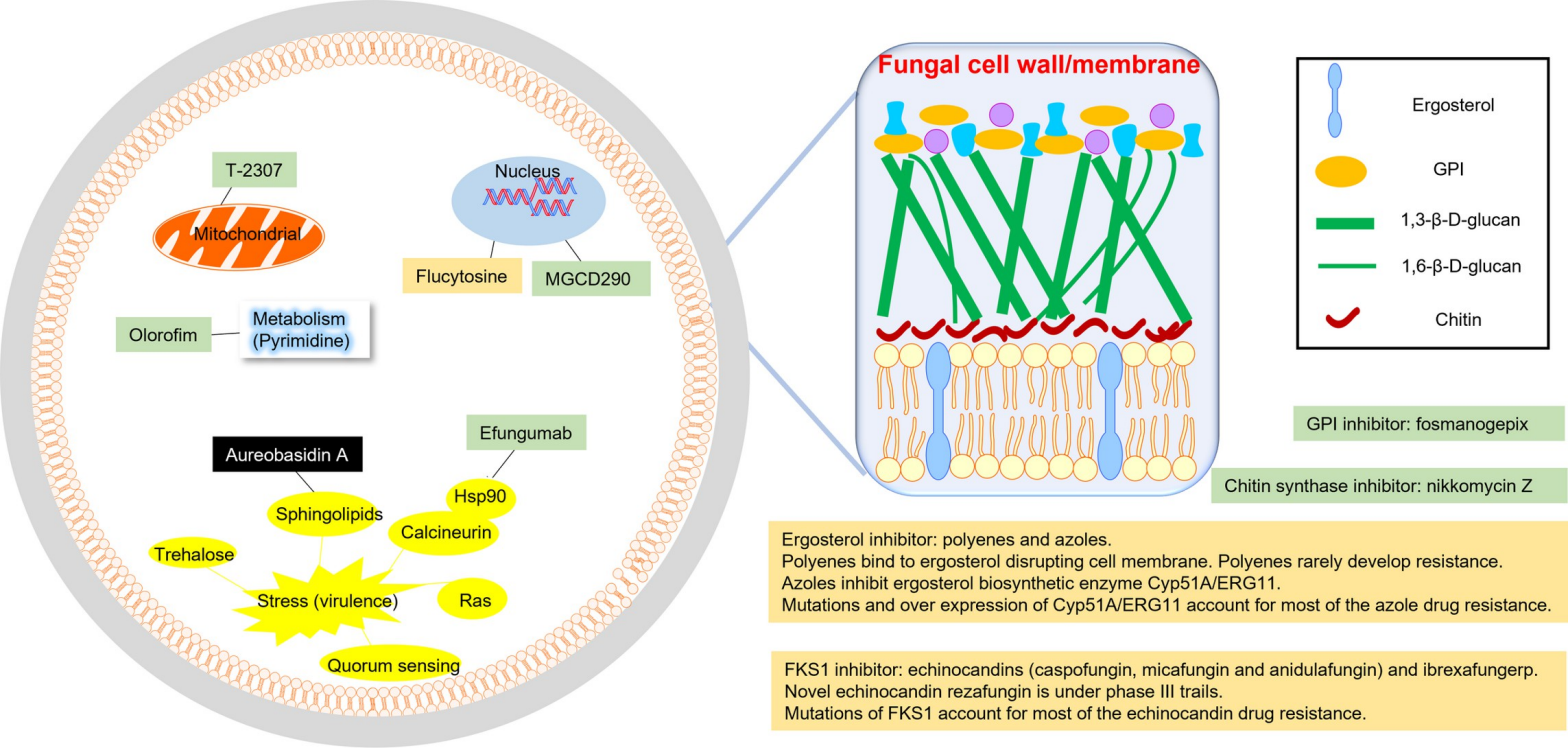
Targets of antifungals. Clinically approved antifungals (gold), antifungals under clinical trials (green), and antifungals in preclinical studies (black).

Most serious fungal diseases are caused by *Candida*, *Aspergillus*, *Cryptococcus*, *Pneumocystis*, and various species of *Mucorales* [1–3]. The current antifungal agents for invasive fungal diseases (IFDs) are limited to 3 classes based on their inhibition targets: ergosterol inhibitors (azoles and polyenes), 1,3-β-D-glucan synthase (GS) component FKS1 inhibitors (echinocandins and the newly approved ibrexafungerp), and flucytosine (often used in combination with polyenes) interfering with RNA and DNA metabolism (Fig 1).

### Ergosterol inhibitors

Ergosterol is the most important sterol of the fungal cell membrane; depletion of ergosterol damages the cell membrane resulting in cell death. **Azoles** inhibit the activity of lanosterol 14α-demethylase enzyme (LDM), which is required for ergosterol synthesis. The off-target effects and the development of drug resistance remain significant concerns of azole antifungals. Recent advances in azole antifungals include the approval of tetrazoles such as oteseconazole by the Food and Drug Administration (FDA), as well as the designation of quilseconazole and VT-1598 as orphan drugs by the FDA [4]. These new tetrazoles have demonstrated significantly reduced off-target effects compared to the previously used triazoles and imidazoles. Furthermore, opelconazole, a first-in-class inhaled antifungal drug belonging to the class of broad-spectrum triazoles, has also received orphan drug designation from the FDA [5]. Inhaled antifungal treatment could be an attractive option due to its potential to shift the risk–benefit ratio of treatment in a favorable direction, considering the adverse effects, problematic drug–drug interactions, and limited exposure of the lung lumen associated with existing oral or systemic antifungal medicines. **Polyenes** including nystatin, amphotericin B, and pimaricin bind to ergosterol, resulting in fungal cell membrane disintegration. Polyenes are rarely associated with drug resistance, but their toxicity and inability to be taken orally remain great concerns. Recently, the nanoparticle-based encochleated amphotericin B (MAT2203), which offers oral availability along with reduced toxicity, is currently undergoing Phase II clinical trials [6].

### GS inhibitors

GS is involved in synthesizing 1,3-β-D-glucan, which is the major component of the fungal cell wall. Since GS and 1,3-β-D-glucan are absent in humans, GS inhibitors interrupt synthesis of the pathogen’s primary cell wall structural polymer without adverse effects or drug–drug interactions associated with other antifungal agents such as azoles and polyenes. **Echinocandins,** including caspofungin, micafungin, and anidulafungin, are the most widely used GS inhibitors [7]; however, they need to be administrated intravenously once daily. Rezafungin is a novel echinocandin with exceptional stability and solubility and a uniquely long half-life allowing for front-loaded drug exposure with once-weekly dosing [5]. The FDA antimicrobial drugs advisory committee recently recommended the approval of rezafungin for the treatment of candidemia and invasive candidiasis in adults. If approved, rezafungin could be the first new *Candida* treatment option in over a decade. The newly approved **ibrexafungerp** also targets GS but with a different mechanistic mode of action [8]. It is worthwhile to mention that ibrexafungerp is not only the first approved first-in-class oral glucan synthase inhibitor, but also the first drug approved in a novel antifungal class in more than 2 decades. Detailed discussions on the mechanisms of the clinically approved antifungals have been well documented by Wang and colleagues [9].

## Other antifungal targets

**Fig 2:**
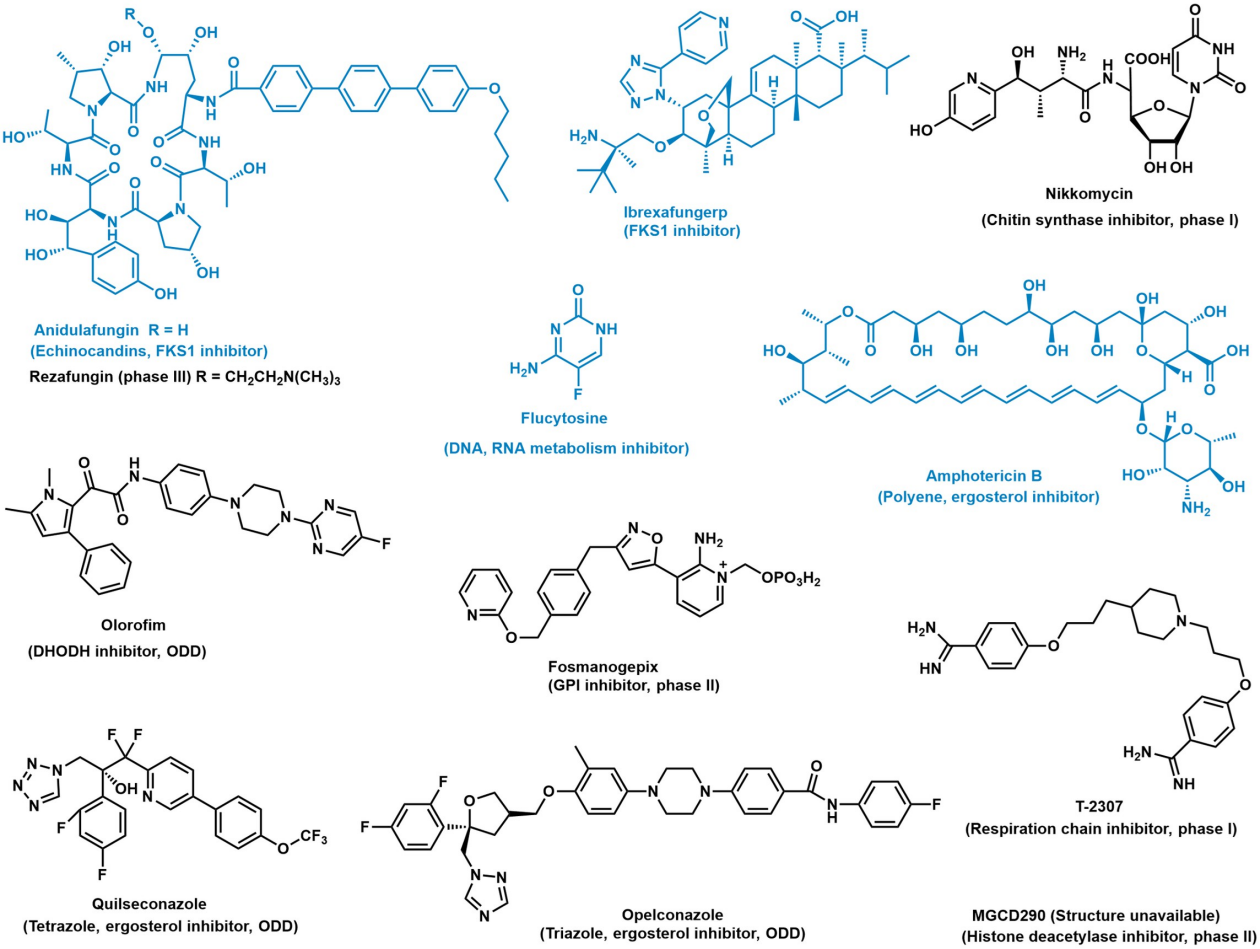
Clinically approved antifungal agents and experimental antifungal agents under clinical trials. Echinocandin class of antifungals is represented by anidulafungin and rezafungin, which displays exceptional stability and solubility. Quilseconazole represents the terazole family of antifungals; opelconazole is the first-in-class inhaled antifungal approved for clinic use. Approved antifungals are in blue, and experimental antifungals in clinical trials are in black. DHODH, dihydroorotate dehydrogenase; ODD, orphan drug designation.

The conventional antifungals are facing serious resistance issues. Antifungal resistance is usually acquired through decreased drug–target interaction [10]. Genetic changes to the target binding site are the major cause of antifungal drug resistance. For example, mutation of genes encoding β-glucan synthase is responsible for most of the echinocandin antifungal drug resistance. Thus, apart from improving antifungals inhibiting the traditional antifungal targets, continued efforts have been made to seek new targets. The new antifungal targets under clinical trials include glycosylphosphatidylinositol (GPI), chitin synthase, histone deacetylase, mitochondrial-related pathways, and pyrimidine synthase (Fig 1). Several promising antifungals against these targets are in clinical trials (Fig 2) [11]. Among them, pyrimidine synthase inhibitor olorofim just received orphan drug designation from the FDA for the treatment of coccidioidomycosis, scedosporiosis, etc. In addition to the advances on established antifungal targets involved in the fungal cell wall/membrane synthesis, and primary cell metabolism, great progress has been made in fungal biofilm inhibition. Andes and colleagues isolated the aromatic polyketide antifungal natural product turbinmicin and found turbinmicin disrupted extracellular vesicle delivery during biofilm growth, which impaired the subsequent assembly of the biofilm matrix [12]. Moreover, basic understanding in fungal virulence is also resulting promising targets for antifungal drug discovery and development. While still in the research and preclinical stages, drugs targeting these novel fungal virulence pathways have great potential of becoming new classes of antifungal.

## Antifungal immunotherapy in invasive fungal infection

The toxicity and increased drug resistance associated with conventional fungal chemotherapies make the search for new antifungal therapies urgent. Immunotherapies such as those targeting the PD-1 and CTLA-4 pathways have revolutionized cancer treatment [13]. Fungal infections can evade the host immunity; thus, it is reasonable to assume that IFDs are amenable to immunotherapy. Unlike traditional chemotherapy, which relies on drugs that directly target the fungus, experimental antifungal immunotherapy works by stimulating the immune system to target and eliminate fungal infection; the immune system has a broader range of targets than any single drug and can adapt to target new or mutated strains of the fungus. Thus, antifungal immunotherapy may be more specific in its action, reducing the risk of off-target effects, drug resistance, and toxicity. The interest in fungal immunology research has increased in recent years, leading to extensive understanding of the cellular and molecular determinants of mammalian antifungal immunity. Fungal immunology and experimental antifungal immunotherapy have been well illustrated in several reviews [14,15]. Antifungal immunotherapies under development include immunotherapeutic vaccines, immunomodulatory drugs, monoclonal antibodies, adoptive T-cell therapy. Of which, an anti-Adh1 monoclonal antibody, Ca37, was demonstrated to be effective against *Candida albicans* and to act together with antifungal drugs to reduce their minimal inhibitory concentrations [16], and the fungal immunotherapeutic vaccine (NDV-3A) for treatment of recurrent vulvovaginal candidiasis is under Phase II trial [17]. The recombinant human granulocyte-macrophage colony-stimulating factor (GM-CSF) has been approved as effective adjuvant therapy for IFDs [18]. Kontoyiannis and colleagues developed the D-CAR T cells, which can reduce the fungal burden of an immunocompromised invasive aspergillosis mouse [19]. In addition, the role of antimicrobial peptides (AMPs) in treating IFDs have been well investigated; for example, peptides HIF-1α and LL-37 can inhibit *C*. *albicans* colonization [20]. Overall, these advances in fungal immunotherapies hold great promise for improving the treatment of fungal infections, especially in patients with weakened immune systems. However, more research is needed to determine the safety and effectiveness of these treatments in humans.

## Perspective

Antifungal therapy development presents more challenges than antibacterial drug development due to the evolutionary conservation between fungal and mammalian cells. Antifungal drugs can target either fungal-specific targets or targets shared with mammals but with structural differences between the two. Thus, understanding the resistance mechanism of current antifungals and in-depth understanding into the pathogenic mechanism of fungal pathogens are essential to identify targets for antifungal therapies with novel modes of action. Other efforts in improving antifungal therapies include the following: (1) repurposing existing drugs: some drugs have intrinsic antifungal activity, such as immunosuppressants FK506 and cyclosporin A; they could be further optimized to treat IFDs; (2) combination therapy: current combination therapy is limited to chemotherapies; combination of antifungal immunotherapy and chemotherapy could increase efficacy and reduce the risk of drug resistance; (3) discovering antifungal natural products by antifungal target gene-directed genome mining: with the basic understanding of antifungal targets, and the development of target genome mining, antifungal target gene-directed genome mining would play an important role in antifungal natural product discovery; (4) leveraging the innate resistance of antifungals in natural host to alleviate clinical antifungal drug resistance; and (5) employing bioengineering and modern synthetic technology to afford antifungal agents diversity. With the development of modern drug discovery technology and extensive investigation of fungal pathogen–host interactions, more antifungal therapies with broad spectrum of action, improved safety profile, and less drug resistance will emerge, especially for the deadly IFDs.
